# Migratory flight behaviour of the pollen beetle Meligethes aeneus †

**DOI:** 10.1002/ps.4550

**Published:** 2017-03-20

**Authors:** Alice L Mauchline, Samantha M Cook, Wilf Powell, Jason W Chapman, Juliet L Osborne

**Affiliations:** ^1^ Department of AgroEcology, Rothamsted ResearchHarpendenUK; ^2^ School of Agriculture, Policy and DevelopmentUniversity of ReadingReadingUK; ^3^ University of ExeterPenrynCornwallUK

**Keywords:** population, migration, pollen beetle, Brassicogethes aeneus, altitude, movement ecology

## Abstract

**BACKGROUND:**

The field ecology of the pollen beetle Meligethes aeneus and its damaging effects on oilseed rape crops are well understood. However, the flight behaviour of M. aeneus, in particular the drivers for migratory movements across the landscape, is not well studied. We combined three established methodologies – suction traps, vertical‐looking radar and high‐altitude aerial netting – to demonstrate that M. aeneus flies at a range of altitudes at different points during its active season.

**RESULTS:**

By linking evidence of high‐altitude mass migration with immigration of pollen beetles into oilseed rape fields, we were able to ‘ground‐truth’ the results to characterise the seasonal movements of this pest across the landscape.

**CONCLUSION:**

We demonstrate that this novel combination of methodologies can advance our understanding of the population movements of pollen beetles and could provide an opportunity to develop predictive models to estimate the severity and timing of pest outbreaks. © 2017 The Authors. *Pest Management Science* published by John Wiley & Sons Ltd on behalf of Society of Chemical Industry.

## 1 INTRODUCTION

The pollen beetle *Meligethes aeneus* (Fabricius) (synonym *Brassicogethes aeneus* Fabricius) (Coleoptera: Nitidulidae) is a small, black, univoltine beetle with a dependence on brassicaceous plant species for oviposition and larval development.^1^, ^2^, ^3^ The introduction of large areas of oilseed rape (*Brassica napus* L.) (Brassicaceae) into the European agricultural landscape in the 1970s was rapidly exploited by *M. aeneus*, and consequential changes in its reproductive success and intraspecific competitive ability^4^ have resulted in its present‐day status as an important economic pest.^5^, ^6^ The migration behaviour of *M. aeneus* is not well studied to date; however, the life cycle and many population parameters are well known.^5^, ^7^ *M. aeneus* beetles are not ground active, so it is assumed that they rely on flight for all dispersal movements across the landscape when in search of host plants for feeding, mating and oviposition. Temperature is a limiting factor on insect flight activity, and recent laboratory studies have shown that their propensity to fly follows a sigmoid temperature–response curve between 6 and 23 °C.^8^ Field studies have recorded solitary flights starting from 10.2 °C^9^ and gregarious flights^10^, ^11^ from 12.3 °C upwards.^12^, ^13^

The main migratory movements of *M. aeneus* across the landscape occur at recognised stages within their life cycle:^1^, ^5^, ^7^

1. On emergence from overwintering sites, adult beetles fly to flowering wild plants to feed on pollen.
2. Adult beetles then migrate to oilseed rape crops to feed and reproduce; firstly to winter‐sown crops in early spring (a high intensity of flights at this time was recorded by Sedivy and Vasak^14^), and then to spring‐sown crops in late spring.
3. New‐generation adults emerge mid‐summer and fly from winter oilseed rape crops that have finished flowering to locate new host plants for feeding; these may include spring brassica crops, but once flowering of these also subsides, the beetles move to forage on pollen from wild plants.
4. As pollen availability and day temperatures fall, the beetles move to overwintering sites.

The rise in temperature in early spring and other meteorological factors^8^, ^15^ trigger migration flights from overwintering sites and also regulate the development of the oilseed rape crop. As a consequence, the timing of the main migration into winter oilseed rape fields usually coincides with the damage‐susceptible early bud stage. Climate‐change‐related variations in air temperature and precipitation are likely to affect crop invasion by pollen beetles, and multimodel predictions by Junk *et al*.^16^ suggest that the onset of crop invasion will be 10 days and 23 days earlier in the near future (2021–2050) and in the far future (2069–2098) respectively. However, the implications for the status of *M. aeneus* as a pest are unclear, as the effect of climate change on the overwintering survival and behaviour of the insect are as yet unknown.

The migratory movements of individuals described above have mostly been inferred from counts of adults on plants throughout the year at different sites, and not from direct recording or tracking of individual flights. Owing to their small size, it has proved difficult to track individuals; however, some studies have been able to estimate the distances flown. Individuals have been recorded travelling 13.5 km using radioactive tracers,^17^ but it is likely that they can travel considerably further.

In addition to the longer migratory flights, *M. aeneus* also perform shorter‐distance, lower‐altitude flights to locate their host plants. The ‘flight boundary layer’ was described by Taylor^18^ as a hypothetical layer of air near the ground within which insects are able to control their movements relative to the ground because their flight speed exceeds wind speed. Taimr *et al*.^17^ showed that *M. aeneus* beetles were able to locate and travel to fields of oilseed rape regardless of wind direction, indicating that they used self‐powered, directed flights within their flight boundary layer, and were recorded at a distance of 300 m from the release point within 2 hours of release. Further evidence from detailed field experiments has confirmed that adult *M. aeneus* use upwind anemotaxis to locate oilseed rape plants.^19^, ^20^, ^21^

To date, little research has focused on the flight behaviour of *M. aeneus* above the flight boundary layer to uncover population‐scale patterns of longer‐distance, higher‐altitude dispersal movements. Therefore, this study investigates the potential for combining data records of pollen beetles from three established recording techniques – suction traps, vertical‐looking radar (VLR) and high‐altitude aerial netting – and combines these data with phenological monitoring of beetles in oilseed rape crops to gain an understanding of their migration movements.

Suction traps collect flying insects by constantly sampling from the air.^18^, ^22^ The Rothamsted Insect Survey operate suction traps that sample at 12.2 and 1.5 m heights, providing a constant, easily comparable measure of insect density at the two heights. High‐altitude insect flights have been studied using scanning radars since 1968.^23^ However, the recent development of automated, vertical‐pointing systems has enabled radar to be used for routine, long‐term monitoring of aerial migration.^24^, ^25^ Vertical‐looking radar (VLR) records information about overflying insects that is related to their speed, direction of movement, orientation, size and shape. Sampling of the aerial fauna at similar heights is required to calibrate the radar records for individual species, and this has been achieved using a balloon‐supported net, which samples the aerial fauna between 180 and 200 m above ground level.^26^, ^27^

Applying this combination of methodologies to advance the understanding of the migratory ecology of *M. aeneus* is novel, and for the first time we aim to ‘ground‐truth’ these approaches by linking evidence of high‐altitude mass migration with immigration of pollen beetles to fields containing oilseed rape. This study was conducted over two years to determine ‘proof of concept’ of this approach for developing an understanding of population dynamics at the landscape scale and the potential for advancing forecasting approaches.

## 2 EXPERIMENTAL METHODS

The aerial netting data were collected in July of 1999 and 2000. All other data (suction traps, VLR and counts on crop plants in the field) were collected from the start of March to the end of August in both 2001 and 2002 and were summed into weekly totals for comparative analyses.

### 2.1 Suction traps

The aerial density of *M. aeneus* was measured daily at two heights – 12.2 and 1.5 m – using the Rothamsted Insect Survey suction trap^28^, ^29^ at Rothamsted (Harpenden, UK). Daily counts of pollen beetles were converted to aerial density in 10^3^ m^3^ air, assuming a 12 h (daytime) period of flight activity, and summed for weekly values.

### 2.2 Diurnal flight activity

A second 12 m suction trap was operated through the summer of 2001 to investigate the diurnal flight activity of *M. aeneus*. The trap was operated from 19 May to 23 August. Insect samples from four time periods were collected separately using a timed bottle changer attached to the suction trap (times shown in BST): ‘Dawn’ 06:00–08:00 h, ‘Day’ 08:00–18:00 h, ‘Dusk’ 18:00–20:00 h and ‘Night’ 20:00–06:00 h.

### 2.3 Vertical‐looking radar (VLR)

The VLR was operated continuously and was also sited at Rothamsted Research; the equipment and operating procedures are detailed by Chapman *et al*.^25^, ^30^ Briefly, the VLR emits a narrow, vertical beam, and overflying insects modulate the radar signal in a way that is related to their speed and direction of movement, their orientation, size and shape. It is capable of detecting individual insects flying within 15 height bands between 150 and 1200 m above the radar, depending on their weight. As *M. aeneus* beetles only weigh between 1 and 2 mg, the radar can only detect this size of insect at the lowest sampling band of 150–195 m above ground level.^25^ Any signal captured within that range was recorded for a 5 min period every 15 min, 24 h a day. The aerial density of overflying insects was found by calculating the volume of air sampled by the VLR for every target, and densities were expressed in terms of the mean number of insects per 10^7^ m^3^ as calculated for each 5 min period. The VLR is unable to identify the species of insect detected using this method alone, so the continual recordings are restricted to specific times during the day and to specific insect weights, to reduce the ‘noise’ from other flying insects. Therefore, the radar data were filtered to daytime records (07:00–19:00 h GMT) of targets between 1 and 2 mg over the sampling period for both years. The aerial densities were then summed to provide the daily density of correct‐sized targets per 10^7^ m^3^ and then corrected to density in 10^3^ m^3^ air to be comparable with the suction trap density data. The aerial densities will be overestimates of true pollen beetle numbers owing to the fact that the VLR will also record other species weighing 1–2 mg, but the data are indicative of periods when lots of pollen beetles are flying.

### 2.4 Collection of migrating pollen beetles using aerial netting

Clarification of the insect species flying at approximately 200 m above the ground was achieved by aerial sampling using a net suspended from a tethered, helium‐filled blimp.^26^, ^27^ The samples were collected at Cardington Airfield (Bedfordshire, UK) on several dates in July of 1999 and 2000 at a variety of times during the day when weather conditions permitted and all *Meligethes* spp. were counted.

### 2.5 Meteorological data

Meteorological data were collected from a meteorological station sited within 20 m of the Rothamsted suction traps. The data were collected every 15 min, 24 h a day, and were combined into daily or weekly means. The variables used were: mean temperature (°C), mean solar radiation (W m^−2^), mean wind speed (m s^−1^), total rainfall (mm) and mean relative humidity (%).

### 2.6 Field assessments

Weekly assessments of the number of *M. aeneus* per plant were conducted over two years in fields of winter (WOSR) and spring oilseed rape (SOSR) on Rothamsted Farm, Harpenden, to ground‐truth the flight data. Four 60 m linear transects were walked in each field (two WOSR and two SOSR fields in 2001; three WOSR and one SOSR in 2002), along which 20 equally spaced oilseed rape plants were sampled for crop growth stage^31^ and the number of *M. aeneus* adults present on the main raceme (on days without rain). Growth stage (GS) was characterised using the BBCH stages described by Lancashire *et al*.,^31^ summarised here as: none (no flower heads), GS 51–57 (green bud), GS 59 (yellow bud), GS 60 (first flowers open), GS 61–63 (early flowering), GS 65 (full flower) and GS 67–69 (flowering declining).

### 2.7 Statistical analyses

#### 2.7.1 Identification of meteorological factors influencing flight at different altitudes

Daily meteorological variables and beetle densities at the three altitudes were compared by calculating Spearman's rank correlation coefficients. The significance levels for rejecting the null hypothesis (of no association between the two variables) based on Student's *t*‐approximation were calculated (see supporting information 1), and the false discovery rate^32^, ^33^ for these comparisons was also calculated to quantify the expected proportion of type I errors.

#### 2.7.2 Correlation between the phenology of the oilseed rape crop and field counts of M. aeneus

The mean number of pollen beetles per plant and the proportion of the crop in flower (GS 60–65) were calculated weekly for all the fields in both years and compared for both winter and spring oilseed rape by calculating Spearman's rank correlation coefficients. The significance of the correlation was determined using a *t*‐test.

#### 2.7.3 Correlation of population flight patterns with immigration and emigration from field crops

Weekly data were divided into periods of immigration and emigration. Immigration was defined as those weeks leading up to and including the maximum number of beetles per plant. The mean field count of *M. aeneus* and the proportion of plants in flower (GS 60–65) for each week during immigration were then compared with the previous week's insect densities at 1.5, 12 and 150–195 m, and correlation matrices were produced using Spearman's rank correlation coefficients. Emigration was defined as the period following the maximum number of beetles per plant in the field, and the data for these weeks were compared with the same week's insect densities, again using Spearman's rank correlation coefficients. These comparisons were chosen to model the movement of insects between flight and the crop.

## 3 RESULTS

### 3.1 Diurnal activity

The total catch of *M. aeneus* from the 12.2 m suction traps is shown in four discrete time periods (Table 1), demonstrating that flight in this species occurs during the daytime.

**Table 1:** Diurnal flight activity of Meligethes aeneus; total numbers caught in a suction trap sampling 12.2 m above ground level within different time periods (May–August 2001)

Sampling period	Times (h BST)	Total *M. aeneus* caught	Mean number caught per sampling hour
Dawn	06:00–08:00	3	1.5
Day	08:00–18:00	211	21.1
Dusk	18:00–20:00	18	9
Night	20:00–06:00	5	0.5

### 3.2 Altitudinal flight profile of M. aeneus

All of the methodological approaches resulted in catches of *M. aeneus*, clearly showing that this species flies at a range of altitudes, up to the highest recordings of 200 m (Table 2). More beetles were caught in the 2002 samples than in the 2001 samples for all methods.

**Table 2:** Altitudinal flight profile of Meligethes aeneus. Summary of total numbers caught using suction traps, vertical‐looking radar (VLR) and aerial netting sampling methods

Sampling method for *M. aeneus*	1999	2000	2001	2002
Suction trap total catch at 1.5 m			136	504
Suction trap total catch at 12.2 m			237	414
VLR records (total number of 1–2 mg insects) at 150–195 m			1916	1500
Aerial netting total catch at 200 m^a^	15	41		

a *M. aeneus* adults were caught in aerial netting samples on seven out of nine sampling days in 1999, and eight out of 11 sampling days in 2000.

**Figure 1:**
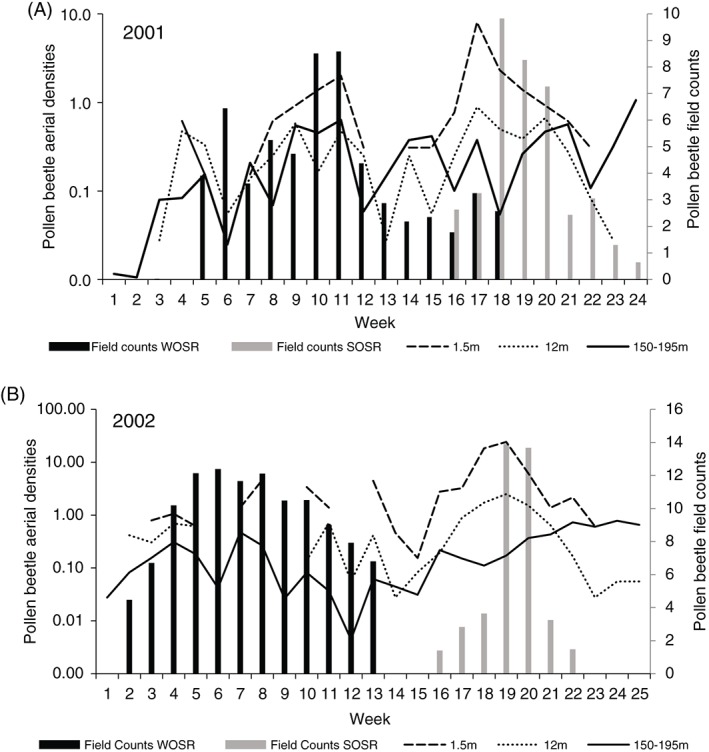
Mean weekly densities of Meligethes aeneus (in 10^3^ m^3^ air) in suction traps at 1.5 and 12 m and VLR at 150–195 m, plotted alongside mean weekly counts of the number of M. aeneus per plant in winter (WOSR) and spring oilseed rape (SOSR) crops in 2001 (A) (12 March–26 August) and 2002 (B) (4 March–25 August).

The average density of *M. aeneus* in flight at 1.5, 12 and 150–195 m was calculated weekly from March to August in 2001 (Fig. 1a) and 2002 (Fig. 1b). Around 3 times more beetles were found in 2002 than in 2001, although both years show similar patterns phenologically. The highest density of beetles occured at 1.5 m, whereas the 12 m and 150–195 m densities were approximately one order of magnitude smaller. Their use of flight at different altitudes varied through the season. Early in the season (March–early April; weeks 1 to 6 in Fig. 1) there was a predominance of flight at 12 m, followed by a period (May to June; weeks 9 to 15 in Fig. 1) where flights occurred at all altitudes. The highest density of beetles flying at any height occurred in July (weeks 18 to 21 in Fig. 1), with by far the most at the lowest altitude, 1.5 m. In August (weeks 22 to 25 in Fig. 1) there was a shift towards high‐altitude flight (at 150–195 m) and the lower‐altitude flights tailed off rapidly.

### 3.3 Identification of meteorological factors influencing flight at different altitudes

Insect density at all altitudes was positively correlated with temperature and radiation while negatively correlated with rainfall and wind speed (which was also found by Skellern *et al*.^21^) (see supporting information 1).

### 3.4 Characterisation of the phenology of the oilseed rape crop and its correlation with field counts of M. aeneus

In both years there was a similar pattern of colonisation by *M. aeneus* in the winter and spring oilseed rape crops. The first few individuals were present from early March in the winter crop from early green bud (GS 50–51) onwards. However, the main population did not arrive in the crops until April when it was approaching flowering (GS 65). The numbers decreased on the winter oilseed rape plants through May and June as the winter crop ceased flowering (GS 67–69). As the new generation of *M. aeneus* emerged in early July, the population reached its annual peak in the spring crop when it was in full flower (GS 65) in 2001, but occurred 2 weeks later in 2002 when the crop was in yellow bud (GS 59), possibly owing to late establishment of the crop. The population again tailed off as the crop finished flowering (GS 67–69).

**Figure 2:**
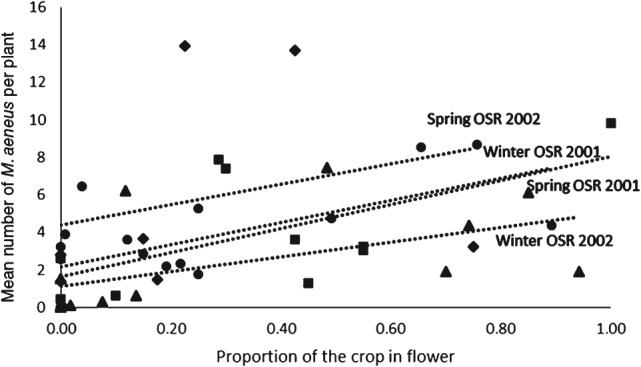
Relationship between the proportion of the crop in flower and the density of Meligethes aeneus in fields containing oilseed rape. Weekly comparisons shown over two field seasons for winter and spring oilseed rape. 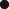 Winter OSR 2001; 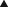 winter OSR 2002; 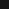 spring OSR 2001; 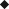 spring OSR 2002.

For winter oilseed rape, the proportion of the crop in flower was positively correlated with the mean number of beetles per plant in both 2001 (*r*_s_ = 0.629, df = 15, *P* = 0.007) and 2002 (*r*_s_ = 0.725, df = 11, *P* = 0.005) (Fig. 2). Spring oilseed rape also showed the same trend, but there were low numbers of replicates; in 2001 the correlation was not statistically significant (*r*_s_ = 0.533, df = 8, *P* = 0.115), and in 2002 there were too few replicates to conduct the Spearman's rank correlation test.

### 3.5 Correlation of population flight patterns with immigration and emigration from crops

Correlation matrices were formed for the period of immigration (S2) and emigration (S3) into and out of winter and spring oilseed rape fields. By focusing on the weeks around the initial colonisation of winter and spring crops in each year (Fig. 1), it is clear that daily patterns of flight are different for the two colonisation periods. Winter oilseed rape colonisation is characterised by low insect densities at 1.5 and 12 m over several weeks, resulting in a gradual build‐up of numbers in the crops. There was a statistically significant, positive correlation between numbers of beetles in the crops and insect densities at 1.5 m (*r*_s_ = 0.638, df = 38, *P* < 0.001), 12 m (*r*_s_ = 0.563, df = 38, *P* < 0.001) and 150–195 m (*r*_s_ = 0.604, df = 38, *P* < 0.001) from the previous week. The first flights follow an increase in daily mean temperatures (*r*_s_ = 0.618, df = 38, *P* < 0.001) and radiation (*r*_s_ = 0.826, df = 38, *P* < 0.001) with low rainfall (*r*_s_ = −0.355, df = 38, *P* = 0.025).

Spring oilseed rape colonisation is characterised by a sharp increase in insect densities at 1.5 and 12 m in early July (week 17 in 2001 and weeks 18 and 19 in 2002), associated with a rapid immigration into the crops. The number of beetles in the crop was positively correlated with the proportion of the crop in flower (*r*_s_ = 0.792, df = 7, *P* = 0.05) and insect densities the previous week at 1.5 m (*r*_s_ = 0.669, df = 7, *P* = 0.05) and 12 m (*r*_s_ = 0.681, df = 7, *P* = 0.05). Specific meteorological triggers are difficult to identify, and the rapid increase in numbers is most likely to be the emergence of the new generation.

Emigration was less well correlated with insect aerial density. As the number of beetles in winter oilseed rape crops declines (seen by the negative correlations with week), there is a correlation with insect density at 150–195 m (*r*_s_ = 0.475, df = 38, *P* = 0.002), but not with densities at any of the other altitudes, while emigration from spring oilseed rape is strongly correlated with insect density at 1.5 m (*r*_s_ = 0.741, df = 9, *P* = 0.009).

## 4 DISCUSSION AND CONCLUSIONS

This study has characterised, for the first time, the altitudinal flight profile of *M. aeneus* throughout their active season. It has provided evidence that this species uses high altitude flights (up to ca 200 m) at specific points during the year, as well as showing multiple periods of low‐altitude flights. It has also been confirmed that there is a strong bias for flight during the day, which could be due in part to their reliance on visual cues during flight^34^ and/or the need to reach the minimum temperature threshold required for flight.^8^ It may also be influenced by the fact that they use plant volatiles for host location and plants release more of these volatiles during the day. These results concur with the findings of Lewis and Taylor,^35^ who found that the peak flight time in this species was 12.44 GMT.

Emergence of overwintered adults (March–early April) is followed by a peak in higher‐altitude flights (at 12 m), which may suggest that medium‐range dispersal movements are occurring from the overwintering site to food sources. Mating occurs on brassicaceous plants from mid‐May onwards,^36^ so dispersal from overwintering sites also ensures mate location and increases population heterogeneity.^37^ The emergence seems to be spread over several weeks that are characterised by warmer weather and low rainfall. These variables have previously been linked with pollen beetle immigration into oilseed crops.^15^, ^16^, ^21^ However, we found that after emergence these meteorological factors have less impact on the timings of flights throughout the rest of the season. Flight in *M. aeneus* was most common at low altitude (1.5 m suction traps), indicating high levels of population redistribution at the local scale. Such dispersal enables the population to relocate across the landscape to find their ephemeral, but highly concentrated host plants.^37^

During the winter oilseed rape crop colonisation period, there is still a considerable level of flight activity, which could be due to continuing emergence from overwintering and/or population redistribution to localised areas of resource availability. Following this period, there is an interesting lull in flight activity that coincides with a drop in the number of beetles in the crops. This occurs in the time between winter oilseed rape flowering and spring oilseed rape flowering, during which time there is no detectable movement of beetles to other food sources. This might indicate that a large proportion of the overwintered adults die at the end of the winter oilseed rape flowering and it is mainly the new‐generation adults that colonise the spring crops. However, it is thought that the new generation does not reproduce in their first year,^36^ yet the spring oilseed rape crops can sustain high levels of oviposition damage;^38^ therefore, some reproductive females must move from the winter to the spring crops.

Immigration of *M. aeneus* into spring oilseed rape is very rapid, and the population reaches its peak in mid‐July, with the arrival of new‐generation adults. The use of low‐altitude flights at this stage is likely to be the most efficient way of quickly locating the nearest resource that is still in abundance at this time. Spring oilseed rape emigration at the end of the active season may be followed by migratory flights at high altitude (150–195 m), as there was evidence of this from the 2001 data (but not 2002, resulting in a non‐significant correlation overall). Utilising the strategy of wide‐range migration prior to overwintering would increase genetic variation in the population in the following year and may increase the geographical range over which the population will emerge. Further investigation into emigration patterns over several years would provide better understanding of this part of the life cycle.

In both years the highest number of beetles making flights at low altitude and subsequently colonising the nearby crops was in early July owing to the population comprising both old‐ and new‐generation beetles. Therefore, timing the sowing of the spring crop to ensure that the vulnerable bud stage does not coincide with this could provide an efficient means to reduce pest damage at this crucial point in crop development.

The combination of sampling techniques used in this study presents a novel approach for understanding population movements of *M. aeneus* at a landscape scale and provides the opportunity to develop predictive models to estimate the size of the following year's population (by comparing the size of the new generation with the overwintered generation the following year). Such ecological understanding would enhance decision support systems such as proPlant expert (http://www.proplantexpert.com) (in the United Kingdom http://www.bayercropscience.co.uk/pollenbeetlepredictor/) that help growers predict the risk period for crop immigration by pollen beetles. proPlant is a web‐based decision support system that has a phenological model of population dynamics of *M. aeneus* that is related to local weather forecasts to predict the start and end of crop immigration as well as major risks of migration events in‐between,^15^, ^39^ enabling more focused monitoring.^40^ The work presented here demonstrates that there is also potential to refine such decision support systems by integrating crop phenology and pest population dynamics. These refined models could provide estimates of pest abundance in addition to forecasts of risk of immigration. With just two years' worth of data, this study highlights the potential of these methodologies for collating a dataset that could be used to refine such models and the value in investing future research effort in this area.

The description of the flight patterns of *M. aeneus* will also be of great importance in implementing integrated pest management control methods such as the push‐pull strategy most effectively.^41^, ^42^ The push‐pull strategy is likely to be most effective at controlling pests during immigration to oilseed rape crops.^38^, ^43^, ^44^, ^45^ Important considerations established from this study include the fact that the immigration pattern is generally linked to the crop phenology, and the arrivals have not always moved from nearby flowering plants, but have potentially flown from long distances at a range of altitudes to reach the crop.

The synergistic output of the novel combination of techniques employed here provides a rationale for long‐term monitoring of the population movements of this pest, and puts value on the modelling of several years' worth of meteorological and biological data potentially to yield specific predictors of immigration to oilseed rape crops.

## Supporting information


**SUPPORTING INFORMATION 1. Correlation matrix for daily densities of Meligethes aeneus (in 10** ^3^ **m** ^3^ air) caught at three altitudes and daily meteorological values (n = 348). Values are Spearman's rank correlation coefficients (r_s_), with significance levels for rejecting the null hypothesis (of no association between the two variables) based on the Student's t approximation (* denotes statistical significance at the 95% confidence level).
SUPPORTING INFORMATION 2. Correlation matrices for the immigration period of Meligethes aeneus into winter (WOSR) and spring (SOSR) oilseed rape crops. Mean field counts of M. aeneus per plant and proportion of the crop in flower correlated with data for the previous week's meteorological values and insect density at three altitudes. Values are Spearman's rank correlation coefficients (rs), with significance levels based on the Student's t approximation. * Denotes statistical significance at the 95% confidence level.
SUPPORTING INFORMATION 3. Correlation matrices for the emigration period of Meligethes aeneus from winter and spring oilseed rape crops. Field counts of the mean number of M. aeneus per plant and proportion of the crop in flower, correlated meteorological values and insect density at three altitudes from the same week. Values are Spearman's rank correlation coefficients (rs), with significance levels based on the Student's t approximation. * Denotes statistical significance at the 95% confidence level.
