# Supplementary material for: Migratory flight behaviour of the pollen beetle Meligethes aeneus
Source: Pest Manag Sci. 2017 Mar 20;73(6):1076–82. doi: 10.1002/ps.4550 (PMC5434912; doi:10.1002/ps.4550)
Supplement: Supplementary file 1 — SUPPORTING INFORMATION 1. Correlation matrix for daily densities of Meligethes aeneus (in 10 3 m 3 air) caught at three altitudes and daily meteorological values (n = 348). Values are Spearman's rank correlation coefficients (rs), with significance levels for rejecting the null hypothesis (of no association between the two variables) based on the Student's t approximation (* denotes statistical significance at the 95% confidence level). SUPPORTING INFORMATION 2. Correlation matrices for the immigration period of Meligethes aeneus into winter (WOSR) and spring (SOSR) oilseed rape crops. Mean field counts of M. aeneus per plant and proportion of the crop in flower correlated with data for the previous week's meteorological values and insect density at three altitudes. Values are Spearman's rank correlation coefficients (rs), with significance levels based on the Student's t approximation. * Denotes statistical significance at the 95% confidence level. SUPPORTING INFORMATION 3. Correlation matrices for the emigration period of Meligethes aeneus from winter and spring oilseed rape crops. Field counts of the mean number of M. aeneus per plant and proportion of the crop in flower, correlated meteorological values and insect density at three altitudes from the same week. Values are Spearman's rank correlation coefficients (rs), with significance levels based on the Student's t approximation. * Denotes statistical significance at the 95% confidence level. [file PS-73-1076-s001.doc]

**SUPPORTING INFORMATION 1**

**Correlation matrix for daily densities of *Meligethes aeneus* (in 10^3^ m^3^ air) caught at three altitudes and daily meteorological values (n=348). Values are Spearman's rank correlation coefficients (r_s_), with significance levels for rejecting the null hypothesis (of no association between the two variables) based on the Student's t approximation (* denotes statistical significance at the 95% confidence level).**

|  | **Density 1.5m** | **Density 12m** | **Density 150-195m** |
| --- | --- | --- | --- |
| **Mean temp** | 0.231 * | 0.187 * | 0.458 * |
| **Radiation** | 0.268 * | 0.287 * | 0.400 * |
| **Rainfall** | -0.106 * | -0.135 * | -0.390 * |
| **% Relative Humidity** | 0.132 * | 0.011 | -0.141 * |
| **Wind speed** | -0.347 * | -0.225 * | -0.733 * |
| False discovery rate | 5% | 6.25% | 5% |

**SUPPORTING INFORMATION 2**

**Correlation matrices for the immigration period of *Meligethes aeneus* into winter (WOSR) and spring (SOSR) oilseed rape crops. Mean field counts of *M. aeneus* per plant and proportion of the crop in flower correlated with data for the previous week's meteorological values and insect density at three altitudes. Values are Spearman's rank correlation coefficients (rs), with significance levels based on the Student's t approximation. * Denotes statistical significance at the 95% confidence level.**

|  | **Mean field counts of *M. aeneus* in WOSR (n=40)** | **Mean field counts of *M. aeneus* in SOSR (n=9)** |
| --- | --- | --- |
| **Proportion of field crop flowering** | 0.809 * | 0.792 * |
| **Density of *M.aeneus* at 1.5 m** | 0.638 * | 0.669 * |
| **Density of *M.aeneus* at 12 m** | 0.563 * | 0.681 * |
| **Density of *M.aeneus* at 150-195m** | 0.604 * | -0.150 |
| **Mean temp** | 0.618 * | 0.200 |
| **Radiation** | 0.826 * | -0.266 |
| **Rainfall** | -0.355 * | 0.067 |
| **% Relative humidity** | -0.710 * | 0.117 |
| **Wind speed** | -0.132 | 0.300 |
| **Week (time)** | 0.806* | 0.788* |
| False Discovery Rate | 5.56% | 12.5% |

**SUPPORTING INFORMATION 3**

**Correlation matrices for the emigration period of *Meligethes aeneus* from winter and spring oilseed rape crops. Field counts of the mean number of *M. aeneus* per plant and proportion of the crop in flower, correlated meteorological values and insect density at three altitudes from the same week. Values are Spearman's rank correlation coefficients (rs), with significance levels based on the Student's t approximation. * Denotes statistical significance at the 95% confidence level.**

|  | **Mean field counts of *M. aeneus* in WOSR (n=40)** | **Mean field counts of *M. aeneus* in SOSR (n=9)** |
| --- | --- | --- |
| **Proportion of field crop flowering** | 0.510 * | 0.501 |
| **Density of *M.aeneus* at 1.5 m** | 0.174 | 0.741 * |
| **Density of *M.aeneus* at 12 m** | 0.103 | 0.583 |
| **Density of *M.aeneus* at 150-195m** | 0.475 * | -0.401 |
| **Mean temp** | -0.061 | -0.519 |
| **Radiation** | -0.016 | 0.246 |
| **Rainfall** | -0.172 | 0.531 |
| **% Relative humidity** | -0.407 * | 0.219 |
| **Wind speed** | -0.007 | 0.246 |
| **Week (time)** | -0.312* | -0.673* |
